# Pro-Con Debate: Nitrous Oxide for Labor Analgesia

**DOI:** 10.1155/2019/4618798

**Published:** 2019-08-20

**Authors:** Manuel C. Vallejo, Mark I. Zakowski

**Affiliations:** ^1^West Virginia University, Morgantown, WV 26506, USA; ^2^Cedar-Sinai Medical Center, Los Angeles, CA 90048, USA

## Abstract

This Pro-Con debate will provide the practitioner with an evidence-based knowledge approach to assist the clinician in determining whether to employ (Pro) or not to employ (Con) this technique in the obstetrical suite for labor analgesia. Nitrous oxide has been used safely in dentistry and medicine for many centuries. However, accumulating preclinical and clinical evidence increasingly suggests previously unrecognized adverse maternal and fetal effects of nitrous oxide, which warrants reconsideration of its use in pregnant women and a more detailed informed consent. Nitrous oxide is associated with metabolic, oxidative, genotoxic, and transgenerational epigenetic effects in animals and humans that may warrant limiting its usefulness in labor. This debate will discuss and review the clinical uses, advantages, and disadvantages of nitrous oxide on occupational effects of nitrous oxide exposure, neuroapoptosis, FDA warning on inhalational anesthetics and the developing brain, research limitations, occupational exposure safety limits, effects on global warming, and potential for diversion.

## 1. Introduction

Inhaled nitrous oxide is the most commonly used labor analgesic in many countries. It is used in greater than 50% of births in Finland, Norway, England, Australia, and New Zealand, 60% of births in the United Kingdom, and 70% of births in Sweden [1–4]. Nitrous oxide analgesia is gaining in popularity in the United States following approval of a delivery system by the Food and Drug Administration (FDA) in 2012 [5]. Nitrous oxide analgesia is self-administered via a commercially available 50% nitrous/50% oxygen intermittent delivery system that produces both anxiolysis and mild analgesia [1, 3]. The pro-nitrous oxide debate will discuss and review the clinical uses and occupational effects of nitrous oxide exposure, neuroapoptosis, FDA warning on inhalational anesthetics and the developing brain, research limitations, occupational exposure safety limits, effects on global warming, and potential for diversion. Recent scientific evidence has found nitrous oxide to be associated with metabolic, oxidative, genotoxic, and transgenerational epigenetic effects in animals and humans that may warrant limiting its use in labor. The con argument develops from the basic science mechanisms of nitrous oxide combined with the known physiologic and biochemical effects associated with its use.

## 2. Pro: Nitrous Oxide for Labor Analgesia

### 2.1. Efficacy of Nitrous Oxide

Access to nitrous oxide can provide more pain management options for parturients who do not have access to an epidural or who cannot or do not want an epidural. Nitrous oxide provides a similar level of pain relief as compared to a paracervical block and opioids but does not have the side effects on the newborn that are seen with injectable opioid medications [1, 5]. Although mothers report nitrous oxide is less effective than an epidural, people who have used nitrous oxide report similar satisfaction levels compared to people who have had an epidural and would use nitrous oxide again if offered in future pregnancies [1, 3, 6].

Nitrous oxide is simple to administer, inexpensive, has not been shown to increase bad health outcomes for mothers or newborns, does not interfere with uterine contraction, and has no adverse effects on the normal physiology and progress of labor [1].

The pain relief with nitrous oxide starts working within a minute, which is less time than for an epidural to become effective. It is also less invasive than using an epidural or injectable opioid. By self-placing the mask, the patient can control her own level of pain relief by choosing when to put the mask on and when to take it off. This can increase the patient's sense of perceived control which can reduce pain perception. Unlike a labor epidural, nitrous oxide allows for strength and freedom of movement. It can also create a sense of pleasure, relaxation, and anxiety relief, allowing the parturient to not care as much about her pain. Also, nitrous oxide use requires the parturient to focus on breathing which may help to explain some of its beneficial effects.

The main drawback of nitrous oxide during labor is that it is less effective than other forms of pain management, including neuraxial analgesia [1–3, 5, 6]. The analgesic efficacy of inhaling a relatively low concentration of nitrous oxide is limited, with few women reporting little or no benefit. Also, it requires repeated self-doses, such that one must hold the mask over the face to provide effective pain management. This could be cumbersome for the parturient who is exhausted or tired and does not want to hold the mask. Reported side effects of nitrous oxide include excessive drowsiness (0-24%), nausea (5-36%), emesis (15%), light-headedness or dizziness (6-23%), a sense of detachment, and claustrophobia from the mask [1–3, 5, 6].

### 2.2. Clinical Uses of Nitrous Oxide in the Labor and Delivery Suite

Nitrous oxide is typically used in the first stage of labor but can also be used for the second stage (pushing, vacuum, or forceps assisted vaginal deliveries), and third stage of labor as well as during postdelivery procedures such as laceration or episiotomy repair, manual removal of the placenta, and uterine curettage [6]. Nitrous oxide is very versatile in the labor and delivery suite; it can be stopped and started at any time, or switched to another method of pain control as needed. Additionally, it can be used before epidural placement if not immediately available or used to supplement an epidural that is not working very well.

### 2.3. Occupational Nitrous Oxide Exposure

The earliest concern regarding the potential adverse effects between nitrous oxide and spontaneous abortion emerged in 1967 when Vaisman [7] reported that 18 of 31 (58%) pregnancies among Russian female anesthetists ended in spontaneous abortion. Of the female anesthetists who had a spontaneous abortion, 14 (78%) used ethyl ether with and without nitrous oxide in the absence of scavenging in the operating room [7]. The average female anesthetist exposure to inhalational anesthetic agents (including nitrous oxide) per week in women with a spontaneous abortion (n=13) was 25 hours or more, compared to less than 15 hours per week in 5 out of 7 female anesthetist with a normal pregnancy suggesting an occupational anesthetic gas exposure effect [7]. The relevance of this study in modern practice is limited due to the current requirement of having effective scavenging required on all delivery systems.

In 1989, Bodin et al. [8, 9] examined the relationship between occupational nitrous oxide exposure and birth outcomes. Their group mailed questionnaires to 3,985 Swedish midwives who were pregnant between 1980 and 1987 that ended up as single births documented in the Swedish Medical Birth Register. After an 84.3% response rate, the pregnant midwives were matched to a control group from the national Swedish register looking for a relationship between occupational exposure in the second trimester of pregnancy and shift work, birth weight, and gestational age at delivery [8]. The only relationship Bodin et al. could find was a significant association between nitrous oxide exposure and reduced birth weight (OR = 1.8). In a follow-up study of fecundity, the same authors divided the nitrous oxide deliveries into five separate categories (0, 1-10 per month, 11-20 per month, 21-30 per month, greater than 30 per month) and found that midwives who reported they assisted at more than 30 deliveries with nitrous oxide use per month had a longer time to pregnancy than those reporting less or no nitrous oxide exposure (fecundability ratio = 0.63; 95% Cl: 0.44-0.95) [9].

Between August 1987 and May 1988, Rowland et al. [10] mailed screening questionnaires to 7,000 female dental assistants registered by the California Department of Consumer Affairs. Of 4, 856 (69%) who completed the survey, 459 reported having become pregnant in the prior four years, and 418 completed a follow-up phone interview. Respondents were assigned to one of five groups: (1) unexposed, (2) low scavenged, (3) high scavenged, (4) low unscavenged, and (5) high unscavenged nitrous oxide exposure, with ≥ 5 hours per week of nitrous oxide exposure being the cutoff point [10]. When scavenging was used to remove excess nitrous gas, there was no reduction in female fertility. Only women exposed to high dose unscavenged nitrous oxide for 5 or more hours per week had a threefold increase in the risk of spontaneous abortion after adjustment for age, smoking status, and exposure to ethylene oxide with a fecundability ratio of 0.41 (95% Cl: 0.23-0.74) [10].

Other studies have failed to demonstrate an association between nitrous exposure and fecundability [11, 12]. Axelsson and Rylander [11] conducted a cross-sectional postal questionnaire study of hospital employees exposed to anesthetic gases and found no difference in the rates of miscarriage among exposed employees (12.4%) and unexposed workers (9.1%), adjusted for age and smoking. In a postal survey and hospital record review of female Danish dental assistants, Heidam [12] determined there was not an increased risk of spontaneous abortion among dental assistants (10.1% exposed vs. 9.5% unexposed).

Provided that nitrous oxide scavenging equipment is used, there is no available data linking nitrous oxide to adverse reproductive effects [13]. In fact, the European Society of Anesthesiology Taskforce in 2015 came out with a position statement in favor of the use of nitrous oxide, stating there is a lack of evidence for a teratogenic effect, reproductive toxicity, increased health hazards and abortion, women exposed or spouses, or men exposed or spouses, and congenital malformation [14].

### 2.4. Nitrous Oxide Effects on Neuroapoptosis

Nitrous oxide induces opioid peptide release in the brain stem leading to the activation of descending noradrenergic neurones, which results in modulation of the nociceptive process in the spinal cord. Several receptor-effector mechanisms including dopamine receptors, *α*2 adrenoceptors, benzodiazepine receptors, and N-methyl-D-aspartate (NMDA) receptors have been implicated although its exact mechanism of action is not known. There are reports of exposure of the developing brain during the period of synaptogenesis to drugs that block the NMDA receptors or drugs that potentiate GABA receptors can trigger widespread neuroapoptosis [15–20]. However, these studies are indirectly relevant at best as methodological details severely limit the extrapolation of these studies' findings to conclusions regarding the potential toxicity of self-administered nitrous oxide during labor. Jevtovic-Todorovic et al. [18] gave 7-day-old infant rats (thought to be the same period of synaptogenesis in human infants) a combination of drugs commonly used in pediatric anesthesia (midazolam, nitrous oxide, and isoflurane) in doses enough to maintain a surgical plane of general anesthesia for 6 continuous hours. They noted that, in the exposed rats, widespread apoptotic neurodegeneration occurred with deficits in hippocampal synaptic function, and with persistent memory/learning impairment [18]. The effect of general anesthesia and sedation on neuroapoptosis may not be a direct result of nitrous oxide alone, but rather an additive or synergistic effect of the result of the combination of these medications on the GABA and NMDA receptors. All previous studies have demonstrated an effect only when used as a component of general anesthesia in combination with inhalational and induction agents [15, 17, 19, 20]. In contrast, Yon et al. [19] observed that in rat pups, aged 1–14 days, who were continuously exposed to 50%, 75%, or 150% nitrous oxide alone for up to 6 hours, nitrous oxide failed to demonstrate apoptotic neurodegeneration. Given the limited and intermittent use of 50% nitrous oxide during labor, total human fetal exposure would be expected to be only a fraction of that produced in the Yon et al. study.

### 2.5. FDA Warning on Anesthetics and the Developing Brain

The FDA has issued a warning reporting repeated or lengthy use of general anesthetic and sedation drugs during surgeries or procedures in children less than 3 years or in pregnant women in their 3rd trimester may affect the development of children's brains resulting in long-term effects on behavior and learning [15]. The warning will result in a labeling change for 11 common general anesthetics and sedative agents that bind to GABA or NMDA receptors, including all halogenated anesthetic gases. Interestingly, nitrous oxide is not on this list. Following the review of all anesthetic agents, the FDA chose not to include nitrous oxide in its warning, which coincides with the current lack of compelling clinical evidence suggesting any significant detrimental effects to either the mother or fetus.

### 2.6. Nitrous Oxide Research Limitations

Most research on nitrous oxide was conducted before scavenging was widely used and many of these were retrospective postal questionnaires [8–10, 21–25]. Furthermore, none of the studies included ambient nitrous oxide level sampling [13, 26]. Self-administered questionnaires have limitations in that they are retrospective, subject to bias, misinterpretation and variation due to the experience, and education of the respondent among other confounding factors [11, 26–28]. Response bias where women who have had trouble conceiving and concerned about occupational exposure may have been more motivated to participate in the survey [10, 11]. A prospective randomized dose response trial measuring the quantity of nitrous oxide in the ambient air and the biological effect of nitrous oxide is needed to precisely characterize the relationship between nitrous oxide exposure and fertility [10, 29].

### 2.7. Nitrous Oxide Occupational Exposure Safety Limits

In order to provide occupational safety and health protection from unacceptable levels of nitrous oxide, the National Institute of Occupational Safety and Health (NIOSH) in 1977 recommended a maximal time-weighted average (TWA) level of nitrous oxide exposure to 25 ppm per procedure over an 8-hour period [27]. The American Conference of Governmental Industrial Hygienists (ACGIH) threshold limit is 50 ppm [30]. In Europe, the limit varies from country to country; for France the limit is 25 ppm, for Italy and Belgium the limit is 50 ppm, and for Germany, Sweden, and the United Kingdom, the limit is 100 ppm [14]. Sweeney et al. [31]. suggested a threshold of 400 ppm nitrous oxide per anesthetic exposure as a result of their investigation on the effects of trace concentrations of dentists who used nitrous oxide for sedation. Yagiela [32] suggests the minimum threshold for biological effects in humans is above a continuous exposure of 100 ppm for 8 hours or 400 ppm per anesthetic administration. Sweeney et al. [31] found that chronic exposure levels of 1,800 ppm of N2O did not exert any detectable biologic effect in humans, suggesting that 400 ppm would be a reasonable exposure level that is both attainable and significantly below the biologic threshold. These levels relate to the biological effect of impaired fertility and fetal toxicity. The lack of quantification of nitrous oxide exposure is a major weakness in previous studies [10, 29]. Both badge dosimetry and an infrared nitrous oxide analyzer are acceptable ways to monitor nitrous oxide levels. Repeated environmental measurements at both Vanderbilt University Hospital and Brigham and Women's Hospital labor and delivery units where nitrous oxide for labor analgesia is used with scavenging have shown nitrous oxide levels to be lower than the NIOSH standard of 25 ppm [33].

### 2.8. Nitrous Oxide Levels in the Dental Office Are Expected to Be Higher Than in Labor and Delivery

Ambient nitrous oxide levels are expected to be higher in the dental office compared to labor and delivery. Nitrous oxide use in dentistry is different than during labor because dentistry uses continuous flow with up to a 70% nitrous mixture, and the patient often exhales near the face of the hygienist and dentist as opposed to the demand valve intermittent flow of 50% nitrous used in labor and delivery. Therefore, the effectiveness of scavenging in the dental office would not be as effective in labor and delivery [13, 27]. Even with this difference of increased ambient nitrous oxide levels in the dental office, there is no available data linking nitrous oxide and adverse reproductive effects if nitrous oxide scavenging equipment is used [13].

### 2.9. Nitrous Oxide Emissions and Its Effect on Global Warming

Aside from individual safety concerns for mother, fetus, and healthcare workers, larger public health aspects of nitrous oxide use during labor should be considered such as nitrous oxide emission and its effect on global warming. Most climate scientists agree the main cause of the current global warming trend is human expansion of the greenhouse effect [34, 35]. There are multiple gases that contribute to the greenhouse effect including water vapor, carbon dioxide, methane, chlorofluorocarbons, and nitrous oxide. The largest human source of nitrous oxide emissions into the atmosphere comes from agriculture accounting for 67%, followed by fossil fuel combustion and industrial processes for 10%, biomass burning for 10%, atmospheric deposition for 9%, and human sewage for 3% [35]. The total greenhouse gas effect of nitrous oxide atmospheric emissions is estimated to contribute less than 0.05% [36]. Clearly, priority would be to concentrate on the major causes of nitrous oxide's contribution to the greenhouse effect in decreasing the effect of global warming. At present, besides global efforts to reduce nitrous oxide waste gas emissions, we are unaware of any commercially available methods to reduce nitrogen oxide emissions from the health care sector.

### 2.10. Nitrous Oxide and Potential for Diversion and Abuse

Another public health risk is diversion, especially by health care professionals who have access and are familiar with its clinical use [37]. Because nitrous oxide is a weak anesthetic, it has limited abuse potential. Nevertheless, acute active inspiration of 100% nitrous oxide can lead to cerebral hypoxemia, asphyxiation and death [37]. Repeated chronic abuse over several months/years can lead to irreversible peripheral myeloneuropathy with the potential for permanent neurologic disability [13, 37]. Nitrous oxide abuse is most prevalent among dentists who incorporate it into their clinical practice [37]. We are however unaware of documented nitrous oxide abuse in the labor and delivery setting. Because of the potential for abuse, the delivery system should be stowed appropriately, and its use regulated in a controlled environment [13].

### 2.11. The Impact of Nitrous Oxide on Methyltetrahydrofolate (MTHFR) Gene Polymorphisms, Homocysteine Levels, DNA Damage, and Neuronal Damage in Primates

Nitrous oxide inactivates B-12, a cofactor for methionine synthetase, which in turn, is responsible for the hepatic synthesis of methyltetrahydrofolate (MTHF) which is necessary for choline synthesis (a component of sphingomyelin), DNA production and subsequent cellular reproduction [38]. It is known that nitrous oxide increases circulating homocysteine concentration and may do so more in patients with variants of the methylenetetrahydrofolate (MTHFR) reductase gene [38]. Nagele et al. [38] set out to determine if patients with MTHFR reductase gene variants have increased cardiac risk from nitrous oxide exposure and whether this can be mitigated by vitamin B12 administration. They determined that, in 500 patients with cardiac risk factors undergoing noncardiac surgery with nitrous oxide, the MTHFR reductase gene variant did not alter homocysteine concentration or the incidence of cardiac injury and vitamin B12, although decreasing homocysteine concentration did not alter the incidence of cardiac injury. Nagele et al. [38] concluded that, based on current evidence, practitioners who feel that nitrous oxide could be beneficial for their patients should not refrain from administering it because of concern for acute homocysteine increase or MTHFR gene variant.

Nitrous oxide can generate reactive oxygen species (ROS). In general, ROS are known to cause DNA or epigenetic modifications [39]. The risk of nitrous oxide and DNA damage (genotoxicity) appears to be of minimal clinical impact and not of significant concern in the perioperative environment. There is some evidence that nitrous oxide is weakly genotoxic (causing DNA damage) in humans, which appears to be similar to that reported for isoflurane and sevoflurane, with no evidence of direct DNA reactivity. Because any potential genotoxic mechanism would have a threshold, it seems reasonable to conclude that neither occasional high exposure to patients as an anesthetic nor low-level exposure to staff within published recommended exposure limits and proper scavenging presents any significant carcinogenic risk [40]. Furthermore, recent investigations conclude little clinical concern of DNA damage in adults exposed to nitrous and data does not exist regarding the clinical implications of the effects of nitrous oxide exposure on fetal DNA [41].

Regarding neuronal damage, Zou et al. [42] exposed postnatal day (PND) 5-6 rhesus monkeys to nitrous oxide (70%) or isoflurane (1.0%) alone, or nitrous oxide plus isoflurane for 8 hours. The brains then underwent pathologic staining for neuronal damage and electron microscopy. They found no significant neurotoxic effects were observed for the monkeys exposed to nitrous oxide or isoflurane alone [42]. However, neuronal damage was apparent when nitrous oxide was combined with isoflurane. There appears to be an augmented effect when nitrous oxide is combined with a halogenated inhalational agent, and that when used alone, is much less likely to cause neuronal damage.

### 2.12. Summary: Pro-Nitrous Oxide for Labor Analgesia

Nitrous oxide has been used safely in dentistry and medicine for centuries and many women who have used nitrous oxide for labor analgesia in the past choose to use it again for subsequent pregnancies [1, 2, 5, 6, 14]. The commercially available 50% nitrous/50% oxygen intermittent delivery system is safe to both mother and baby and is an acceptable alternative for analgesia for all stages of labor and postdelivery procedures [1, 2, 5, 6, 13, 14, 29, 36, 43].

## 3. Con: Nitrous Oxide for Labor Analgesia

### 3.1. Analgesic and Psychologic Effects

Proponents tout the analgesic effect of nitrous oxide as comparable to narcotics and with high satisfaction [1, 5]. However, labor pain scores were no different in a randomized double blind study comparing air to nitrous oxide, and a recent study revealed a median pain score change of zero following nitrous oxide [44, 45]. Nitrous oxide functions as a dissociative anesthetic [46]. Women rated nitrous oxide as less effective pain relief than epidural analgesia but satisfaction scores were similar [47].

The medicalization of modern birth has produced ‘push back', with women desiring a more ‘natural' birth experience, with fewer medications and interventions [48]. Patient goals include maintaining a sense of control, a degree of mobility and self-determination of the degree of pain relief desired [49]. Nitrous oxide as a self-administered noninvasive alternative may serve a subset of women who may have opted to forgo neuraxial analgesia. The availability of nitrous oxide during labor did not change the neuraxial utilization rate [50].

### 3.2. Occupational Exposure and Safety Limits

Chronic occupational exposure to nitrous oxide causes spontaneous abortions and reduced fertility in humans [9, 10, 21]. NIOSH developed safety limits for nitrous oxide in 1977 and last reiterated in 1994 [51]. However, nitrous oxide limits (25 ppm OSHA) were not based on scientific proof of safety but rather at what level would decrease audio-visual performance [30] and with a 50 ppm limit to prevent CNS impairment and embryo-fetal damage [52]. European countries have similar or higher nitrous oxide exposure limits, ranging from 25 ppm (France), 50 ppm (Italy, Belgium) to 100 ppm (Germany, Sweden, UK), [14]. Although a 2015 European expert opinion stated nitrous oxide was a safe anesthetic [14], the task force was funded by a manufacturer of nitrous oxide (Air Liquide, France) and expert opinion which was formed before more recent evidence about NMDA-receptor related molecular damage and learning disabilities became available [53–55]. In 2016 and 2017 the FDA issued Drug Safety Communication alerts concerning the adverse effect of anesthetics including N-methyl-D-aspartate (NMDA) antagonists on the brain during rapid synaptogenesis, advising to defer elective surgery requiring general anesthesia until after 3 years age [15, 56]. Although nitrous oxide was not specifically listed by the FDA, likely due to lack of compelling direct clinical evidence suggesting that it poses a significant risk, nitrous oxide is a well-known NMDA antagonist and thus in theory may pose similar risks. A 2019 European review discusses the safety of nitrous oxide use and occupational exposure risk, while acknowledging historical concerns [57]. Several articles express concern regarding occupational exposure of waste anesthetic gases and provide data showing increased markers of DNA damage, oxidative stress, and genetic instability [58–60].

### 3.3. Metabolic Disruption and Physiologic Effects of Nitrous Oxide

**Figure 1:**
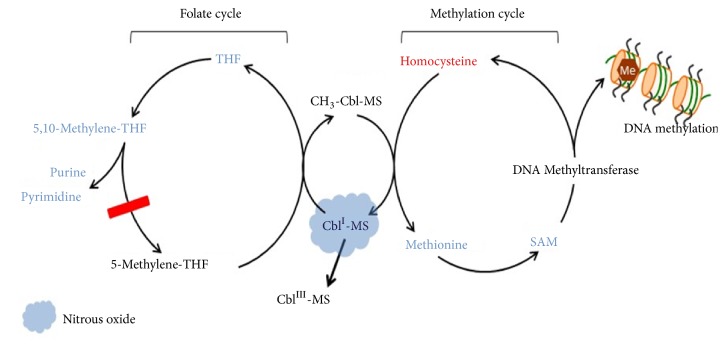
Nitrous oxide effect on methionine synthase and folate metabolic cycle. Chan MT, Peyton PJ, Myles PS, Leslie K, Buckley N, Kasza J et al. Chronic postsurgical pain in the Evaluation of Nitrous Oxide in the Gas Mixture for Anaesthesia (ENIGMA)-II trial. Br J Anaesth. 2016:117:801-11.

Nitrous oxide interferes with methionine synthase which performs a critical role at the junction of two key metabolic cycles, the folate cycle and the recycling of homocysteine to methionine (converts to S-adenosylmethionine, a required methyl donor and DNA methylator) (see Figure 1). Nitrous oxide inactivates methionine synthase by reducing cobalt, permanently inactivating vitamin B12, a required cofactor [61].

#### 3.3.1. Folate Cycle: DNA Thymidine Effect

Blocking the folate cycle affects biosynthetic pathways including de novo thymidylate (dTMP) synthesis and de novo purine synthesis [62]. This leads to inadequate 5, 10-methylene tetrahydrofolate, and inadequate cytosol dTMP synthesis thus allowing uracil misincorporation into DNA in the nucleus (T->U substitution error). 5-methylTHF accumulates 4-fold in the nucleus, suppressing dTMP synthesis and causing DNA damage [63]. Preexisting low Vitamin B12 levels and/or folate depletion exacerbate these metabolic downstream effects of nitrous oxide including DNA double strand breaks and may result in apoptosis of the rapidly dividing cell [64]. In normal rats, a 60 minute exposure to 50% nitrous oxide was associated with abnormal thymidine synthesis [65]. Nitrous oxide is not directly carcinogenic but there is some evidence it is weakly genotoxic in humans [40]. One study found no effect of nitrous oxide combined with desflurane on DNA or redox status for 1.5 h exposure, although the authors commented on the unintended low power of their study [41].

#### 3.3.2. Methylene Tetrahydrofolate Reductase (MTHFR) Enzyme

MTHFR enzyme mutations decrease methionine synthase efficiency and dTMP synthesis. Nitrous oxide at 66% for 2 hours during surgery in healthy adults increased plasma 5-methylTHF by 20% (indicating folate trapping), decreased Vitamin B12, and increased homocysteine levels by 22% in wild type MTHFR, increased by 76% in MTHFR 1298CC genotype and 36% in 677TT genotype [64]. A 2-hour exposure to nitrous oxide significantly reduced vitamin B12, a 0.6-1.3 hr. exposure significantly decreased serum folate and reduced vitamin B12 in humans, which can predispose to neurological side effects [66–68]. Human mutations of the MTHFR enzyme increases plasma homocysteine, exacerbating nitrous induced metabolic changes [64]. In contrast, Nagele et al. determined nitrous oxide exposure in patients with MTHFR mutations did not significantly affect the incidence of cardiac injury and the authors concluded practitioners should not refrain from administering nitrous oxide [38]. In the accompanying editorial, Myles points out the Nagele study was not adequately powered to detected differences in myocardial infarction, and that the incidence of myocardial infarction was 6% in the placebo group and 2.8% in the B-vitamin group (P=0.09), with a lower rate of study drop-out in the nonnitrous group, a potential confounder to a negative study [69].

#### 3.3.3. Methionine Cycle and Elevated Homocysteine

Methionine synthase inactivation interferes with converting homocysteine to methionine. The associated increase in plasma homocysteine may affect coagulation, endothelial adhesion, alters vascular response and remains elevated for days [70, 71]. Nitrous oxide 4-hour exposure in humans significantly increased plasma homocysteine by 10 micromolar (P<.004) to 22.6 ±11.4 micromol/L. [72]. In humans randomized to nitrous oxide (~3 hour) exposure during surgery homocysteine levels increased the duration and relative risk of myocardial ischemia by factor of 1.9, P<.05 [73]. Hyperhomocysteinemia increases cardiac risk by causing acute increases in endothelial dysfunction, platelet aggregation and a procoagulant effect [73]. Acute increase of plasma homocysteine from an oral methionine load was associated with ‘substantial impairment of endothelial function in healthy human' in a significant linear inverse fashion, P<.001, with an 85% reduction in mean flow mediated dilatation at 2 hr. [74]. Acute hyperhomocysteinemia significantly impaired coronary flow velocity reserve at 4 hr, P<.002, and caused a 15% decrease in average diastolic peak velocity, P<.002 [75]. These effects should be avoided in pregnancy, especially in women with possible vascular or perfusion related issues such as intrauterine growth retardation or hypertensive disorders like preeclampsia, who already have endothelial dysfunction and elevated cardiovascular risk. Elevated homocysteine was associated with higher odds of preeclampsia and prematurity with homocysteine levels >15 microM/L associated with an increased chance for abruption [76]. In contrast, several studies highlight the safety of nitrous oxide administration in patients undergoing major surgery with known or suspected cardiovascular disease [77–79].

### 3.4. Nitrous Oxide Effects on NMDA-Receptor Antagonism and Neuroapoptosis

Nitrous oxide is associated with cellular damage and cell death in the rapidly growing brain when combined with halogenated inhaled anesthetics or when administered in hyperbaric concentrations (150% nitrous oxide) [70]. In a rat model of exposure at the peak of synaptogenesis, postnatal day 7, the combination of anesthetics nitrous oxide 75%, isoflurane 0.75% and midazolam 9mg/kg for 6 hours resulted in a tenfold increase in reactive oxygen species (ROS) in neurons, lipid peroxidation, mitochondrial injury, deletion of neurons, with long-term cognitive impairment and delayed learning [80, 81]. This model used anesthetics producing both NMDA antagonism and GABA agonism. These changes could be prevented by a ROS scavenger or compound that restores mitochondrial integrity. Mitochondria can be damaged by increased calcium ion influx and production of ROS leading to apoptosis. A pure NMDA antagonist, MK-801, alone caused significant neuronal apoptosis in a dose dependent manner, starting at 4-hour exposure and increasing thereafter in rats [82]. Rat fetuses exposed to NMDA antagonism at term also had significantly increased neuronal apoptosis in the dentate gyrus, hippocampus and ventromedial hypothalamus [82]. Observed neuronal damage with NMDA antagonism alone are similar to those discussed earlier with mixed NMDA antagonism/GABA agonism in the rat model [80, 81, 83, 84]. Recent investigations have focused on anesthesia induced oligodendrocyte apoptosis [53]. Long-term derangement of myelination may also produce long lasting neural changes [85].

#### 3.4.1. NMDA Antagonism in Nonhuman Primates

NMDA antagonism initiates events leading to neuronal cell damage or death. NMDA receptors are widely distributed, especially in the nervous system and in the rapidly developing brain (third trimester, 2nd year of human life). NMDA antagonism for 4-6 hours induced cell death for nonhuman primate and rodent brain cells [86, 87]. In Rhesus Macaque monkey at postnatal day 5-6, NMDA antagonism (ketamine infusion) produced severe neural cell death in the neocortex after 9 hours (P<.05) and a 58% loss of neuronal cells in culture after 6 h exposure [86, 88]. Following 5 h NMDA antagonism with ketamine, neuroapoptosis in the frontal cortex increased 4-5 fold (P<.003) in the rhesus macaque third trimester fetus and newborn, P<.003 [87]. Fetal neurons were 2.2-fold more susceptible to apoptosis then neonatal neurons. NMDA-receptor antagonism induced apoptosis is exposure time and age dependent; apoptosis started to increase after 4-hour exposure, while no changes occurred after postnatal day 25 (rapid synaptogenesis is over) in the rhesus [87–89]. Rhesus macaque monkey and humans have similar distributions of NMDA receptors in the brain. Interestingly, nitrous oxide alone in the rhesus monkey did not produce neurotoxic effects, while the combination of nitrous oxide in addition to isoflurane did produce neuronal damage [42].

Nitrous oxide at 60% produces NMDA-receptor blockade has postsynaptic effects and has an onset faster than ketamine [90, 91]. NMDA antagonism significantly increased oxidized DNA, neuronal apoptosis and DNA breaks on COMET assay by 1.8-3 fold, P<.05 [92]. Neurotoxicity could be prevented by using glutamate or calcium free solution in cell cultures, free radical scavenging, NR1 antisense (prevents increased NR1 expression), or SN-50 nuclear factor kB translocation inhibitor.

### 3.5. ROS and Oxidative DNA Damage

Almost all exposure to anesthetic gases (e.g., sevoflurane, isoflurane, and nitrous oxide) increases DNA damage. Nitrous oxide decreases expression of antioxidant enzymes like glutathione peroxidase, leaving DNA more susceptible to damage from ROS [61]. In a meta-analysis, exposed individuals had evidence of increased DNA damage as reflected by lymphocyte cytokinesis block micronucleus assay, P<.0001 [93, 94]. Residents in anesthesiology and surgery had increased DNA damage on COMET assay, decreased antioxidant defense by lower glutathione peroxidase, superoxide dismutase and catalase, with an increase in proinflammatory IL-8, P<.001 [95, 96]. Oxidative DNA damage and oxidative stress markers (8-isoPGF2, TBARs) are associated with nitrous exposure and not with sevoflurane or isoflurane; path analysis was significant for nitrous producing ROS, and ROS then producing DNA damage (P<.05) [61]. Nitrous oxide 70% for about 3 hours during surgery in humans significantly increased DNA damage 2-fold and correlated with postoperative wound infection [97]. Recent investigations conclude there is little concern for DNA damage in adults exposed to nitrous oxide, although there are no human data regarding the effects of nitrous oxide exposure on fetal DNA [40, 41].

### 3.6. Informed Consent on Second-Hand Exposure Risk

Institutions may bear liability for second-hand exposure risks for staff and visitors. Human congenital anomalies were increased in female nurses in British Columbia during 1990-2000 with exposure to nitrous oxide associated with an OR 1.82 for all anomalies and OR 3.2 for integument anomalies [98]. Of note, only 67% of the maternity units reported having modern scavenging systems. The authors stated “…risks may be present with mean exposure below NIOSH exposure guidelines.” Recently, nitrous oxide use in labor resulted in exposures exceeding NIOSH recommendations 40-68% of the time [99]. Nitrous oxide exposures should be monitored in the labor rooms to ensure adequate scavenging and room air exchange flow for the safety of patients, visitors and health care personnel. Australian veterinarians had a 2.5-fold increase risk of preterm labor for exposure to >1hr/week of unscavenged anesthetic gas [100]. Anesthetists with Glutathione S-transferase T1 genetic variants had a significantly higher rate of DNA damage (P<.05) during occupational exposure to general anesthetics including nitrous oxide [101].

### 3.7. Fetal Effects of Maternal Nitrous Oxide

Nitrous oxide is a relatively insoluble inhaled anesthetic that rapidly crosses the placenta. A 1-3 hour exposure inactivates methionine synthase in the mother* and* fetus [102, 103]. Nitrous oxide use by parturients ranging from minutes to 11 hours revealed human placental methionine synthase activity decreased, with a faster decrease in women with lower vitamin B12 levels [104]. Over 20% of women may be deficient in vitamin B12 at term, exacerbating the effects of nitrous oxide exposure [102]. After a 1-hour exposure to 50% nitrous, methionine synthase activity in the fetal rat liver was 18% of baseline [103]. Methionine synthase activity in human liver was 50% after 46 min of exposure to 70% nitrous, and 0% after 200 min of exposure [103]. Recovery of methionine synthase activity may take up to 3-4 days. The fetal effects of maternal nitrous oxide administration may warrant maternal and fetal testing for predisposition to its adverse metabolic effects. The neonatal effects of in utero exposure to nitrous oxide is unknown, however Apgar scores and umbilical blood gases are unchanged, with no known clinical adverse effects [105]. The neonatal effects of decreases methionine synthase activity are unknown at this time.

#### 3.7.1. Epigenetic Effects

Nitrous oxide exposure may also have epigenetic and transgenerational effects. Nitrous oxide and isoflurane exposure in a rat model caused substantial epigenetic modulation downregulated expression of brain-derived neurotrophic factor (BDNF) and c-Fos within 2 h [106]. MK-801, an NMDA antagonist, caused phosphorylation of histone H3 and epigenetic changes within 30 min in rat prefrontal cortex [107]. Ketamine, another NMDA antagonist, also affected epigenetic histone modifications in a rat model [108]. Nitrous oxide may decrease serum vitamin B12 and folate acutely. Prolonged Vitamin B12 and folate shortage was associated epigenetic changes including altered cardiometabolic risk factors in human offspring [109]. Nitrous oxide generates ROS, which are known to cause DNA damage or epigenetic modifications [39].

#### 3.7.2. Potential Consequences of Nitrous Oxide on Human Behavior and Cognition

Multiple studies have shown an association and/or causation of general anesthetics with neuronal apoptosis and learning disabilities in fetal and neonatal rats, nonhuman primates and humans [15, 18, 53, 80, 110, 111]. Basic science evidence shows the ability of general anesthetics, NMDA antagonists and nitrous oxide to produce neuronal and oligodendrocyte apoptosis, metabolic derangements or genotoxicity in mother and fetus. Exposure to anesthesia as an infant may have induced apoptosis of myelin producing oligodendrocytes with a decrease in white matter brain volume on MRI in children age 12-15 years old, P=.016 [112].

As little as 90-120 min of total exposure time to general anesthetics from different episodes was associated with an increased incidence of learning disability and ADHD in young human children (adjusted hazard ratio 1.8, P<.04) [54, 55]. Multiple exposures to general anesthetics in young children was significantly associated with learning disabilities and attention-deficit/hyperactivity disorder, hazard ratio 2.17 (95% CI, 1.32-3.59) with decreases in cognitive ability and academic achievement [55]. We cannot predict which neonates or infants exposed to nitrous oxide during labor will need anesthesia following unexpected NICU admission or surgery in the period of increased susceptibility to neuroapoptosis (third trimester, 3 years), nor do we currently know if there are any potential effects of exposure to intermittent 50% nitrous oxide/50% oxygen during labor.

### 3.8. Informed Consent

Informed consent must include discussion of the possible risks of nitrous oxide on maternal and fetal health, including prolonged effects beyond the anesthetic period [102]. The risks and benefits must be discussed relative to each patient's medical history and relative contraindications (See Table 1). Maternal consent for nitrous oxide may include “…some animal studies have shown effects on animal babies and it is not known if in the future there may be proven negative effect on human babies”[47]. Staff and visitors on Labor and Delivery may also need to be informed about the potential adverse effects of nitrous oxide exposure. State regulations require notifying visitors and staff (e.g., California Prop 65), specifically listing nitrous oxide as a teratogen (e.g., New Jersey) or listing nitrous oxide as having reproductive toxicity with no known safe limits [113–115]. The FDA warnings in 2016 and 2017 amplify public awareness and concerns [15, 56]. Delay of elective surgery requiring general anesthesia during rapid neural synaptogenesis (<3 years old) has been suggested [16, 56]. Nitrous oxide use during labor conforms with a personal choice for elective use of an anesthetic, not a necessity required by urgent medical need.

### 3.9. Summary: Con Nitrous Oxide for Labor Analgesia

Nitrous oxide can produce metabolic, oxidative, apoptosis and epigenetic changes in the mother and fetus. Use of any anesthetic, especially nitrous oxide in the parturient, requires medical evaluation and decision making. Relative contraindications include parturients and/or their fetuses with metabolic, genetic and/or cardiovascular risk factors, which affects more than a third of all pregnant women. If used, exposure time to nitrous oxide should be limited, less than 3-4 hours (associated with the start of neuronal cell death for NMDA antagonists) and probably to less than 1 hour (maternal and fetal methionine synthase inactivation). Staff and visitors may be at exposure risk which also imposes legal/regulatory burdens. Why use an ineffective pain reliever in an elective situation with known potential adverse effects for both parturients and fetuses? It is certainly no laughing matter and not part of a ‘natural' birth!

### 3.10. Conclusion

Nitrous oxide has been used safely for labor analgesia over 100 years and many women choose to use it again in a later delivery [1, 2, 5, 6, 14]. Nitrous oxide 50% /oxygen 50% delivery system is safe to both mother and baby and is an acceptable alternative for analgesia at all stages of labor and/or postdelivery procedures [1, 2, 5, 6, 13, 14, 29, 36, 43]. However, the last 20 years of scientific evidence has found nitrous oxide to be associated with metabolic, oxidative, genotoxic, and transgenerational epigenetic effects in animals and humans that may warrant limiting its use in labor.

## Figures and Tables

**Table 1:** Nitrous oxide relative contraindications during pregnancy.

Condition		Prevalence
Metabolic Risk		
	Low vitamin B12	29%
	Low folate	6.4%
	Hyper Homocysteinemia	21%

Genetic Risk		
	MTHFR homozygous polymorphisms 677C>T, 1298A>C	20% western Europe
	Non-Hispanic white CDC	22%
	Non-Hispanic black CDC	4.6%
	Mexican American CDC	23.4%
	Fetal MTHFR polymorphism	Consider parental heritage

Cardiovascular risk		
	Preeclampsia spectrum	5-10%
	Hypertension 20-44 y.o.F	10.4%
	Hypercholesterolemia 20-44 y.o. F	9%

Drugs of Abuse (NMDA Antagonists) history		
	Ethanol use pregnancy CDC	10%
	Phencyclidine/hallucinogen	0.5%
	Nitrous oxide general population	4-8%
	Nitrous oxide UK	Second to cannabis

Medical condition		
Closed space (nitrous causes expansion)	e.g. COPD, asthma attack, occlusion of middle ear e.g. infection	

## Additional Points

*Highlights. *(i) Nitrous oxide can be an effective and useful alternative to labor epidural analgesia. (ii) Nitrous oxide is safe for the parturient and the fetus according to clinical evidence. (iii) Nitrous oxide can be used for all stages of labor including postdelivery procedures. (iv) Nitrous oxide is an NMDA antagonist with the potential for neurotoxic effects. (v) Nitrous oxide affects two basic metabolic cycles, folate and methionine. (vi) Nitrous oxide rapidly crosses the placenta and can affect both maternal and fetal/neonatal methionine synthase. (vii) Nitrous oxide can generate reactive oxygen species which may cause genetic instability.
